# Hyperlipidemia attenuates the mobilization of endothelial progenitor cells induced by acute myocardial ischemia via VEGF/eNOS/NO/MMP-9 pathway

**DOI:** 10.18632/aging.204314

**Published:** 2022-10-03

**Authors:** Jidong Zhou, Hang Li, Liying Xun, Lei Wang, Qitao Zhao

**Affiliations:** 1 School of Pharmaceutical Sciences, Shandong University of Traditional Chinese Medicine, Jinan 250355, China; 2 School of Traditional Chinese Medicine, Shandong University of Traditional Chinese Medicine, Jinan 250355, China; 3 R&D Department, Hubei Minkang Pharmaceutical Group Co. Ltd., Wuhan 430040, China

**Keywords:** hyperlipidemia, endothelial progenitor cells, mobilization, acute myocardial ischemia, VEGF/eNOS/NO/MMP-9 pathway

## Abstract

This study aims to explore the role of hyperlipidemia in the mobilization of bone marrow (BM) endothelial progenitor cells (EPCs) induced by acute myocardial ischemia (AMI). To establish the hyperlipidemia complicated with AMI (HL-AMI) model, SD rats were intragastrically administered the high-fat emulsion for 4 weeks. Then their left anterior descending arteries were ligated. Rats in each group were randomly subdivided into seven subgroups. During 1st ~ 7th day following AMI modeling, rats in 1st ~ 7th subgroups were selected to be phlebotomized from their celiac artery after being anesthetized by pentobarbitone in turn. The quantity of circulating EPCs (CEPCs) was detected by flow cytometry, the expression of VEGF, eNOS, NO, MMP-9 in myocardial tissue was analyzed by western blot, and their plasma level was assayed by ELISA. Dynamic curves were plotted using these data. Within 7 days following AMI, compared with the AMI rats, in the HL-AMI rats, the myocardial infarct size, the plasma activity of CK, CK-MB, and the collagen deposition all remained at the higher levels; meanwhile, these rats showed more significant decreases in the count of CEPCs, the plasma level of VEGF etc., and their expression in myocardial tissue (*P* < 0.05 or *P* < 0.01). Our study showed that hyperlipidemia may attenuate the mobilization of BM EPCs induced by AMI via VEGF/eNOS/NO/MMP-9 signal pathway, which might partly account for hyperlipidemia hampering the repairs of AMI-induced cardiac injury.

## INTRODUCTION

Myocardial ischemia and resultant heart failure are common symptoms of various cardiovascular diseases (CVDs), which are the leading cause of death worldwide. It is reported that hyperlipidemia patients have worse prognosis after acute myocardial ischemia (AMI). A study aimed to assess 5-year outcomes after AMI in a large multicenter cohort of patients showed that, in hypercholesterolemia patients, the risk of long-term mortality and cardiovascular events is twice as high than the control patients [1]. However, the underlying mechanism remained unclear.

The ability of adult human heart to generate new cardio myocytes and vascular endothelial cells is limited, thus failing heart could be repaired by bone marrow (BM)-derived stem/progenitor cell-based cardiac regeneration after AMI [2–4]. Endothelial progenitor cells (EPCs) play vital roles in revascularization and tissue repairs [5]. In adult animals, EPCs are generally stored in BM, and in a resting state under physiological conditions. Only a small fraction of these cells develops into the circulating endothelial progenitor cells (CEPCs). To repair injured tissues, BM EPCs must be mobilized to the peripheral blood, and then home in the damaged site [6–8]. AMI is the most well-known acute pathological stimulus for the mobilization of BM EPCs [6, 9], which depends on the activation of matrix metalloproteinase 9 (MMP-9) and endothelial nitric oxide synthase (eNOS) with various mobilizing factors, including vascular endothelial growth factor (VEGF) and nitric oxide (NO) [8, 10].

It has been shown that hyperlipidemia may lead to CVDs via impairing the quantity and the function of EPCs, including attenuated the adhesion, the migration, and the tube formation of this kind of cells, and accelerated its senescence [11, 12]. But available research data cannot answer the following questions, whether hyperlipidemia could inhibit the mobilization of EPCs induced by AMI or not, and whether this negative regulation could partly account for the cardiac injury deteriorated by hyperlipidemia following AMI. The present study is designed to figure out the answers.

## MATERIALS AND METHODS

### Reagents

Phycoerythrin (PE)-conjugated anti-CD34 monoclonal antibody was obtained from Santa Cruz Co., USA (sc-7324). Mouse anti-rat GAPDH monoclonal antibody, FITC-labeled anti-vWF antibody, rabbit anti-rat MMP-9 monoclonal antibody, and HRP-labeled goat anti-mouse polyclonal IgG were purchased from Abcam, UK (ab9484, ab8822, ab76003, ab6789). Red blood cell lysate was obtained from BD Co., USA (349202). Enzyme-linked immunosorbent assay (ELISA) kits for detecting the plasma level of MMP-9, NO, eNOS and VEGF were purchased from Beijing Sizhengbai Technology Co., China (CRE0026, CRE0110, CHE0115, CRE0010). The high-density lipoprotein cholesterol (HDL-C), low-density lipoprotein cholesterol (LDL-C), total triglyceride (TG), total cholesterol (TC) ELISA kits, and 2,3,5-triphenyltetrazolium chloride (TTC) were purchased from Nanjing Jiancheng Co., China (A112-1-1, A112-1-1, A112-1-1, A111-1-1, D025-1-3). Masson’s trichrome stain kit was purchased from Beijing Solarbio Science & Technology Co., China (G1340). Total protein extraction kit was bought from Applygen Technologies, China. Propylthiouracil and bile acid sodium were purchased from Sigma-Aldrich, USA (191813, T0750). Enhanced chemiluminescence reagent was purchased from Millipore Co., USA (WBKLS0500). Cholesterol was obtained from LifeSpan BioScience Co., USA (RS0423B4013J). RIPA lysis solution was purchased from Beyotime Institute of Biotechnology, China (P0013B). All other reagents were ultrapure grade.

### High fat emulsion preparation

High fat emulsion (HFE) was prepared according to the literature with some modifications [13]. Cholesterol 80 g, sodium deoxycholate 16 g, and methylthiouracil 8 g were added in sequence to melted lard 160 g in a 2000 mL beaker and thoroughly mixed. Tween-80 160 mL was added to the mixture. In the meantime, propylene glycol 160 mL and distilled water 240 mL were added to another 500 mL beaker, and mixed at 60°C on an electric oven. Subsequently, the two components were mixed to prepare the HFE, which was stored at 4°C.

### Animals

Experiments were conducted on male SD rats (SPF, 150–160 g) obtained from the Lukang Animal Experimental Center (SCXK (LU) 20170001, Shandong Province, China). All animal experiments were performed in accordance with the Regulation of Animal Care Management of the Ministry of Public Health, People’s Republic of China (document No. 55, 2001), and were approved by the Ethics Committee of Shandong University of Traditional Chinese Medicine (approval number: DWSY201610227). We made every effort to minimize the distress and number of animals used.

### Experimental procedure

Our experimental design is shown in Table 1. In our research group, the success rate for getting hyperlipidemia complicated with AMI (HL-AMI) rat model was 80.2%. So, to make sure that the number of rats in each group met the statistical analysis requirements at the end of the experiment, 280 rats were enrolled at the beginning. At the end, 56 rats per group, 8 rats per subgroup were reserved.

**Table 1:** Experimental procedure +: rats were sacrificed and phlebotomized, and FCM was performed to quantify the number of CEPCs in their peripheral blood.

**Day/groups (number)**	**Ctrl (*n* = 56)**		**AMI (*n* = 56)**		**HL (*n* = 56)**		**HL-AMI (*n* = 56)**	
Day 1–13	NS administration				HFE administration			
Day 14	Collected blood from their inner canthus and measured their plasma lipid level etc.							
Day 14–20	NS administration				HFE administration			
Day 21	Collected blood from their inner canthus and measured their plasma lipid level							
Day 22–28	NS administration				HFE administration			
Day 29	Sham operation		Coronary artery ligation		Sham operation		Coronary artery ligation	
Day 30–36	**Time points at which the CEPCs content and the plasma level of VEGF etc., were evaluated in each subgroup rats**							
	**Subgroup**	**Day 30**	**Day 31**	**Day 32**	**Day 33**	**Day 34**	**Day 35**	**Day 36**
	1	**+**						
	2		**+**					
	3			**+**				
	4				**+**			
	5					**+**		
	6						**+**	
	7							**+**

At the beginning of this study, 280 rats were enrolled to experiment. The success rate for getting hyperlipidemia model, AMI model was 91.4%, 87.8% respectively, the total success rates for getting HL-AMI model was 80.2%. So, at the end of experiment, 56 rats per group, 8 rats per subgroup were reserved.

After adapting to the environment for one week, the 280 rats were randomly divided into the following four groups, with 70 rats per group, the control group (Ctrl), the hyperlipidemia group (HL), the AMI group (AMI), and the HL-AMI group. The HL and HL-AMI groups were intragastrically administered the HFE (10 mL/kg/day) once a day for 4 weeks, during which, the Ctrl and AMI groups were administered saline solution (NS). Based on their blood lipid levels, the rats failing to meet the hyperlipidemia criteria were weeded out.

Then, the AMI and the HL-AMI groups underwent coronary artery ligating operation. The Ctrl, the HL groups were subjected to the same surgical procedure, except for the coronary artery ligation. Rats met the criterion of AMI model were entered into the next step.

According to the previous method [8], in order to investigate the mobilization of EPC, rats in each group were randomly subdivided into seven subgroups, with 10 rats per subgroup. At serial time points after the HL-AMI models (on day 1–7 following AMI modeling, or day 30–36 after HFE administration), rats in subgroups 1–7 were anaesthetized with pentobarbital and phlebotomized from their celiac artery in turn. CEPCs in whole fresh blood samples from each rat were quantified by flow cytometry (FCM). To show the change in the content of CEPCs during the 7 days, dynamic curves were plotted using the FCM data.

### Hyperlipidemia animal model

To induce the hyperlipidemia model, rats were intragastrically administered the HFE (10 mL/kg/day) once a day for 4 weeks. On day 14 and 21 following HFE administration, rats were anaesthetized with ether. Venous blood was collected from their inner canthus, and the plasma levels of TC, TG, HDL-C, and LDL-C were measured by ELISA. Rats with severe hyperlipidemia, defined as TC ≥ 3.0 mmol/L, TG ≥ 2.5 mmol/L, LDL ≥ 1.50 mmol/L, and HDL ≤ 0.50 mmol/L, were classified as the hyperlipidemia model. The plasma lipids data collected on day 21 showed that, the success rate for getting hyperlipidemia model achieved 91.4%.

### AMI model

**Figure 1:**
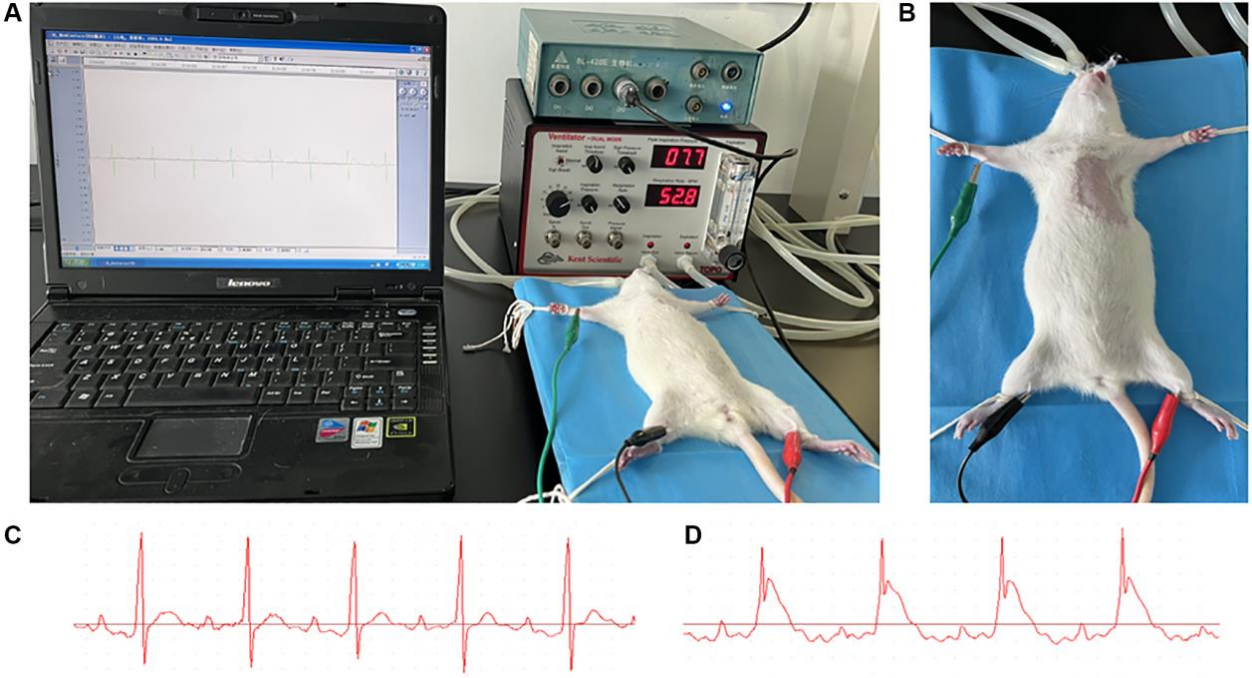
**AMI model preparation.** (**A**) After anaesthetized with an intraperitoneal injection of 2% pentobarbitone (0.2 ml/100 g), the rat was placed on a temperature control board for the surgical procedures. An intubation cannula connecting with a volume-controlled ventilator was plugged in trachea from its mouth. Three electrodes were subcutaneously placed into the three limbs of rat, and connected to a multi-channel recorder by which the electrocardiogram was recorded. (**B**) The fur at the left chest was shaved and the skin disinfected with 75% alcohol. (**C**, **D**) The echocardiography of rat before and after the coronary ligation; the ST segment elevation could be observed.

The rat AMI model was prepared as previously reported [8]. As shown in the image below (Figure 1), the rat was anaesthetized with an intraperitoneal injection of 2% pentobarbitone 0.2 ml/100 g, and fixed on the operating table for the surgical procedures. An intubation cannula connecting with a volume-controlled ventilator was plugged in trachea from its mouth. Three electrodes were subcutaneously placed into the three limbs of the rat, and connected to a multi-channel recorder by which the electrocardiogram was recorded. After the fur was disinfected with 75% alcohol, the left chest was opened between the 3–4 intercostal space. The left anterior descending artery (LAD) was ligated by a 6–0 silk suture 2 mm below the tip of the left atrial appendage. Successful ligation was verified by the color change of hearts and the ST segment elevation in the echocardiography. The heart was repositioned to the chest. After the blood and fluid were absorbed with sterilized absorbent cotton, the chest cavity was squeezed closely. The muscles and skin were sutured layer-by-layer gradually. The skin at the suture area was disinfected with iodophor. The endotracheal intubation, three electrodes, and the string mentioned above were removed. The rat was placed on the right side on a temperature control board, and was put into a disinfection cage after it was awake. After surgery, each rat was intramuscularly injected with penicillin 100,000 units per day for one week.

The critical period is 24 hours after surgery, during which few rats may die. Animals that pass this period generally survive for a long time. In our study, the success rate for getting AMI model achieved 87.8% (112 out of 128).

### Myocardial ischemic area analysis

The myocardial ischemic size was measured according to Sang’s method [14]. After phlebotomized from their celiac artery, the heart of the rats was taken out immediately. From the apex to the base, it was cut into 0.1 cm slices, which were placed in 1% TTC solution (prepared with PBS) for 10 minutes at 37°C. Then, slices were placed in 4% formalin solution for 30 minutes. Normal myocardium is stained red, the infarcted one is not stained, and displayed a pale color. At the end, these slices were quantitatively analyzed by pathological image analysis system.

### Myocardial fibrosis analysis

To characterize collagen fibers in myocardial tissue in the 7th subgroup rats of each group, their formalin-fixed and paraffin-embedded myocardial tissue sections were stained using a masson’s trichrome stain kit; by which collagen is stained blue, nucleus is dyed black; cytoplasma and muscle fibers are red. The process of operation follows the protocol described in the kit instructions.

### Flow cytometry analysis of EPCs

EPCs are cells positive for endothelial markers, such as VEGF receptor 2 (VEGFR2), and hematopoietic stem cell markers, such as CD34 [5, 15]. We used immunofluorescent staining for CD34 and the VEGFR2 to identify mononuclear cells (MMNs) positively in blood as CEPCs. We incubated whole fresh blood samples anticoagulated with EDTA.2Na with PE-conjugated anti-CD34 antibody and FITC-conjugated anti-VEGFR2 antibody to identify the CD34^+^/VEGFR2^+^ cells among the MNCs. We dissolved the red blood cell lysate in distilled water and applied it to the cells at a final concentration of 10% (v/v).

To count the EPCs per milliliter of blood, two-color cytometry analysis of the samples was performed on a cytometer equipped with a four-color option (BD FACS Jazz). EPCs were detected by gate analysis excluding events with different origins, such as non-specific staining events and non-hematopoietic circulating cells. The data were collected from 100,000 cells for each sample and analyzed with BD FACS software.

### Western blot analysis

Hearts were removed for protein extraction after rats were phlebotomized from their celiac artery. The left ventricular tissue sample was taken under ligature at 2 mm and stored at −80°C before use. Total protein was extracted according to the manufacturer’s instructions of the total protein extraction kit. The concentration of protein was determined by the BCA protein assay kit.

The protein was separated by 10% SDS-PAGE and transferred to nitrocellulose membranes. The membranes were blocked by 5% skim milk powder diluted in PBS with 0.05% tween (PBST; pH 7.6), and were incubated overnight on a rocking platform at 4°C with antibodies against MMP-9 (1:2000), eNOS, VEGF and GAPDH (1:5000). Then, the membranes were washed with 50 mM PBST, and incubated with the secondary antibodies for 1 h at room temperature, and then washed thoroughly. The proteins on the membrane were visualized by enhanced chemiluminescence solution. The membranes were exposed with an imaging system (Western Blot Workflow System, Bio RAD, USA) and the relative intensities of protein bands were analyzed by Image Lab (Bio RAD).

### ELISA assay

According to the manufacturer’s instructions, the content or activity of MMP-9, NO, eNOS VEGF, CK, CK-MB in the plasma of rats were measured by ELISA. The whole fresh blood samples were centrifuged for 15 min at 3000 rpm. The supernatant was stored at 4°C. Samples were tested at the same time after all the rats had been sacrificed and phlebotomized. The plasma levels of VEGF etc., are expressed as nanograms per milliliter, units per milliliter, nanomoles per milliliter, or millimoles per milliliter.

### Statistical analysis

The continuous variables are presented as mean ± standard error of the mean (SEM). We used the category variables to estimate the statistical significance by one-way ANOVA and post hoc Bonferroni test using SPSS Statistics version 19.0 (IBM, USA). A *P* value of less than 0.05 was considered statistically significant.

### Availability of data and materials

The datasets generated during and/or analyses during the current study are available from the corresponding author on reasonable request.

## RESULTS

### Change in the content of blood lipid in each group during 7 days after AMI

**Figure 2:**
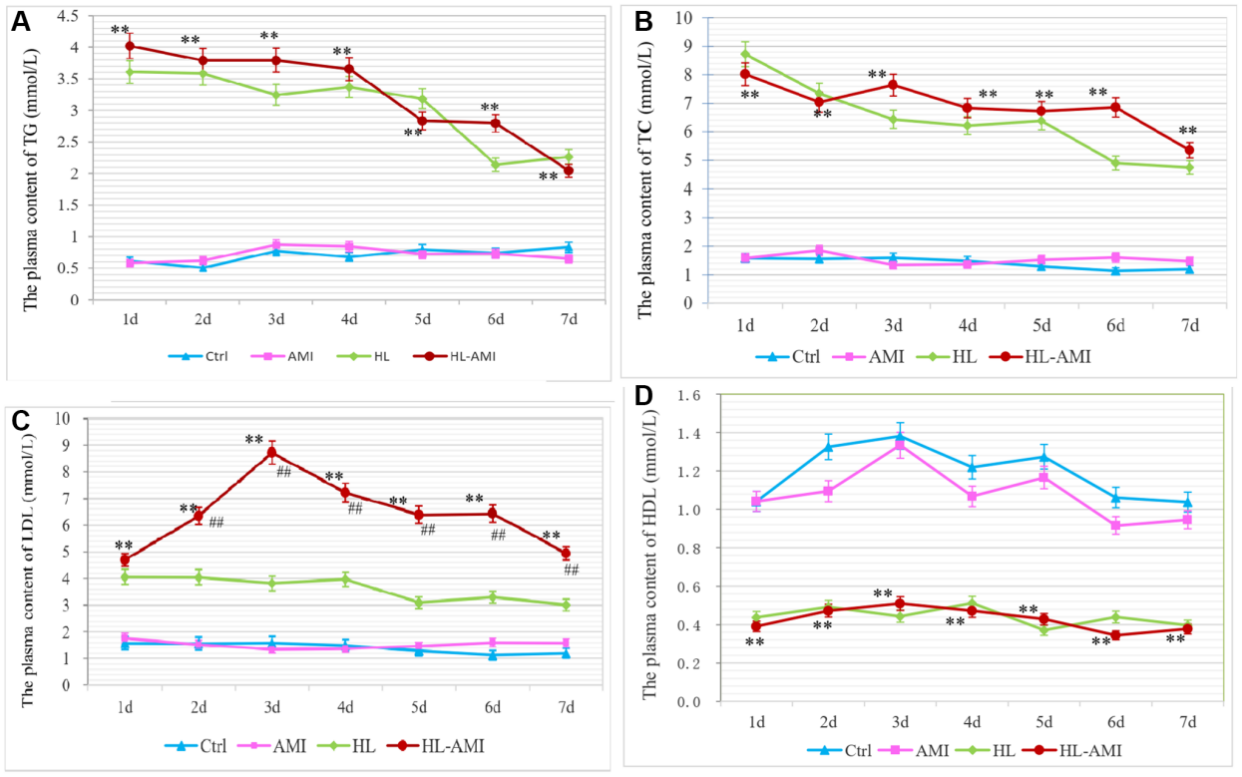
**Change in the plasma content of TG, TC, LDL-C and HDL in each group rats during 7 days after AMI.** (**A**–**D**) The plasma concentration of TG (**A**), TC (**B**), LDL-C. (**C**) and HDL-C (**D**) in rats of the Ctrl, HL, AMI, and HL-AMI group respectively. ^*^, ^**^, *P* < 0.05 or *P* < 0.01, compared with the AMI group. Each data point represents the average of eight rats in one group, and vertical lines indicate the SEM.

The plasma concentration of TG, TC, LDL-C and HDL in each subgroup of rats was shown in Figure 2. Within 7 days following AMI operation, in the Ctrl and the AMI group, the blood content of above four kinds of lipid all fluctuated at the normal level, and there were no significant differences between the two groups (Figure 2A–2D, *P* < 0.01).

During the same phase, in the HL and HL-AMI groups, the plasma concentration of TG, TC, LDL remained at the abnormal higher levels, while the content of HDL-C fluctuated at the aberrant lower line. Due to the cessation of HFE administration, their blood levels tended to decrease. The changes pattern of the plasma content of TG and TC were similar in these two groups, which decreased gradually day by day (Figure 2A, 2B, 2D, *P* < 0.01). However, in the HL-AMI group, the plasma concentration of LDL-C displayed the more complicated changes model. This value upregulated rapidly, reached its peak on day 3, then decreased gradually (Figure 2C, *P* < 0.01).

### Hyperlipidemia hindered the repairmen of cardiac injury induced by AMI

To evaluate the cardiac injury, on the 4th day after the LAD ligation (or in the 4th subgroup of each group), the myocardial ischemic area was verified by TTC staining; and the serum activity of myocardial enzyme was detected by ELISA kits. Moreover, to assess the deposition of collagen on the 7th day following AMI, the myocardial tissue sections of the 7th subgroup rats were performed masson staining.

**Figure 3:**
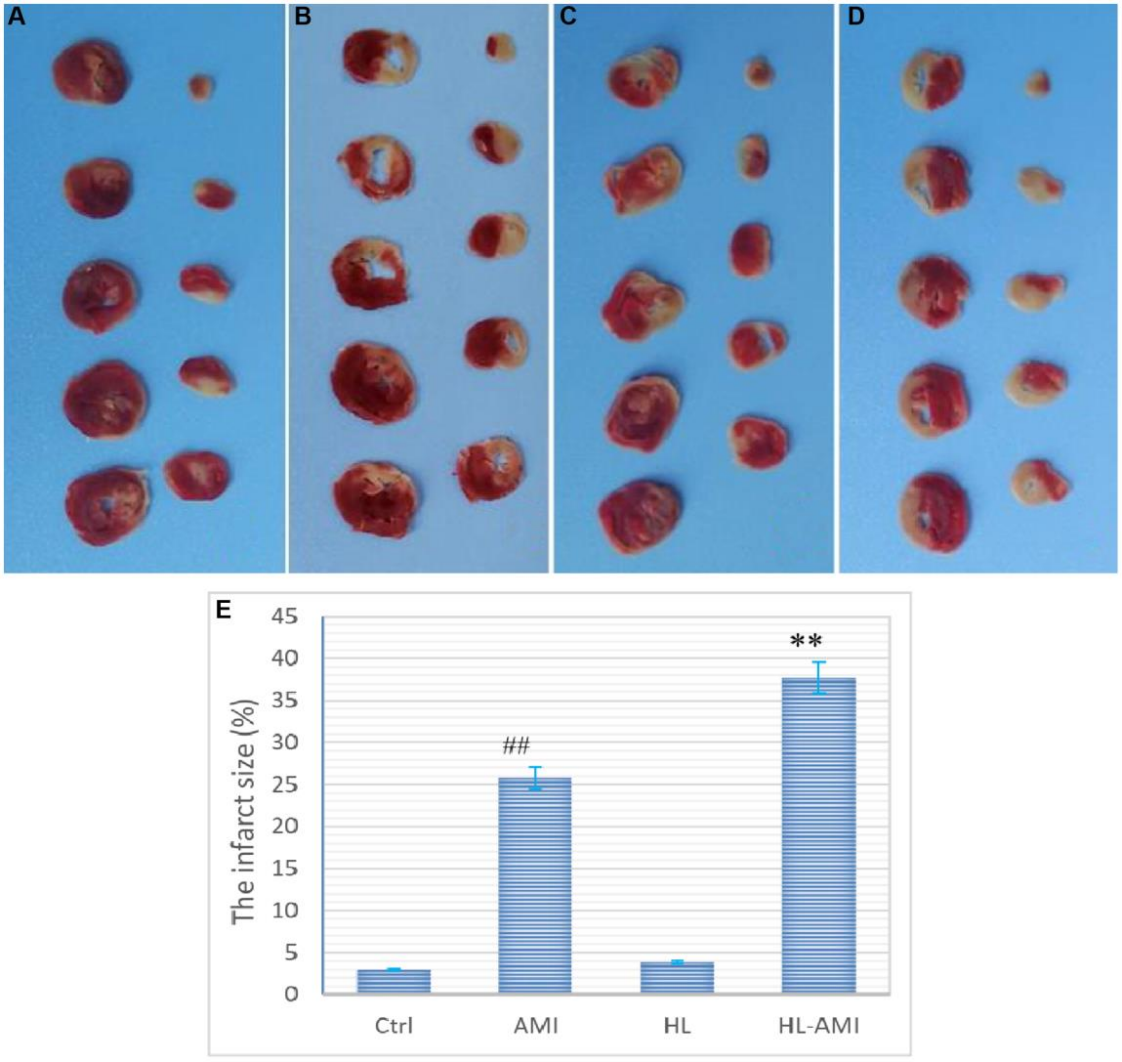
**Myocardial ischemic area in the 4th subgroup rats of each group.** (**A**–**D**) (represents the Ctrl, HL, AMI, and HL-AMI group, respectively), Photos of myocardial sections stained by TTC, viable tissue was stained red and infarct area was pale and unstained; (**E**) Diagram of the myocardial infarction rate of the four groups. MI infarct size was expressed as the percentage of the infarct area relative to the whole myocardium. ^**^, ^##^, *P* < 0.01, compared with the AMI, the Ctrl group, respectively. Data are expressed as mean ± SD (*n* = 8 animals in each group), and vertical lines indicate the SEM.

As shown in Figure 3, the viable tissue was stained red and the infarct area was pale and unstained (Figure 3A–3D). In the HL-AMI group, the infarct size was significantly higher than that size in the AMI group (37.68 ± 4.4% vs. 25.81 ± 3.01%, Figure 3E, *P* < 0.01).

**Figure 4:**
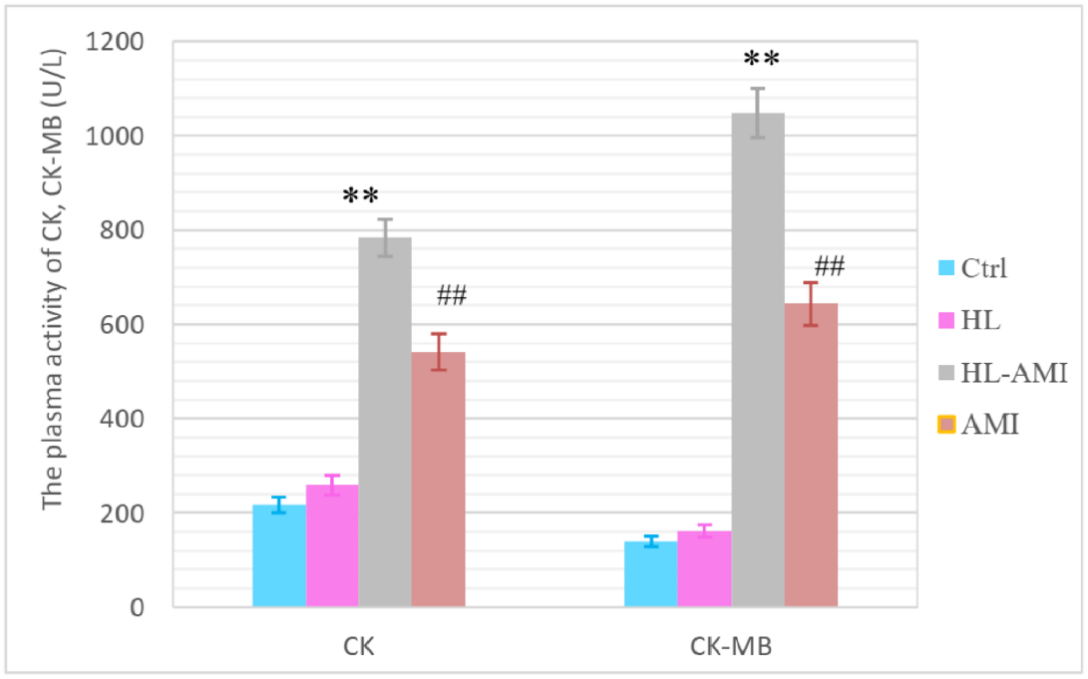
**The plasma activity of CK, CK-MB in the 4th subgroup rats of each group.** According to the manufacture’s instruction, the CK, CK-MB plasma activity were analyzed by ELISA. ^**^, ^##^, *P* < 0.01, compared with the AMI and the Ctrl group respectively. Each data represents the average of eight rats in one group, and vertical lines indicate the SEM.

The serum activity of myocardial enzyme is a sensitive index of myocardial damage. As shown in Figure 4, on the 4th day after AMI, among the four groups, the blood activity of CK, CK-MB in the HL-AMI group remained at the highest level, which was significantly higher than that activity in the AMI group, the second highest one (Figure 4, *P* < 0.01).

**Figure 5:**
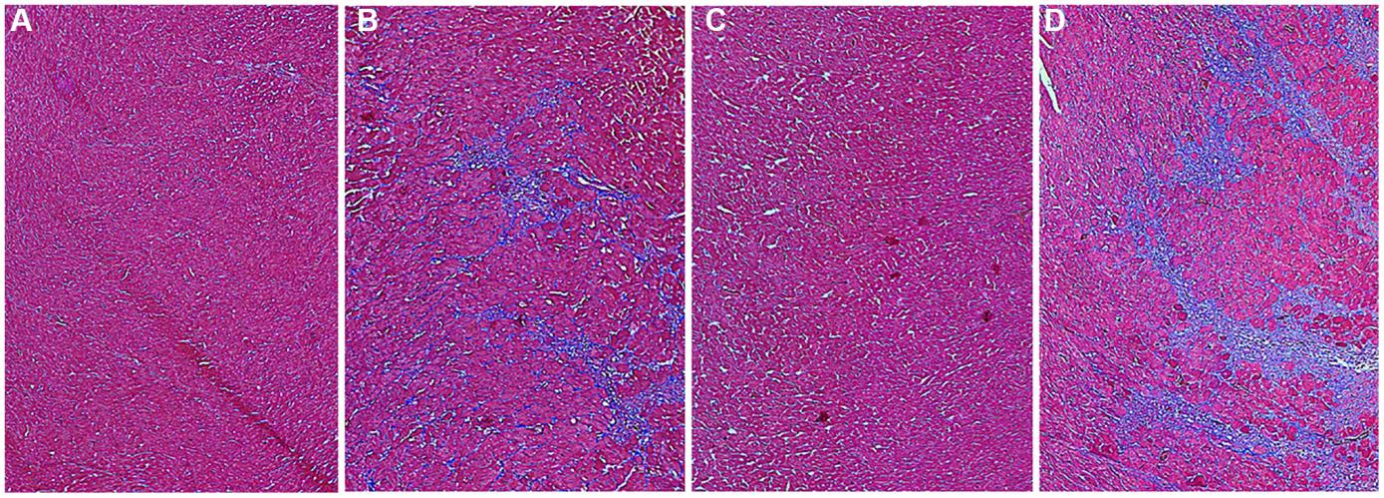
**The collagen deposition of myocardial tissue in each group rats on the 7th day after AMI.** To characterize myocardial tissue damage, the formalin-fixed and paraffin-embedded myocardial tissue sections were stained using a masson’s trichrome stain kit; by which collagen is stained blue, nucleus is dyed black; cytoplasma and muscle fibers is red. Stained sections were observed under optical microscopy, and were photographed by digital camera. (**A**–**D**) Photos of myocardial sections stained by masson G in rats of the Ctrl, HL, AMI, and HL-AMI group, respectively (×100).

On the 7th day after AMI, the collagen deposition of myocardial tissue in each group was showed in Figure 5. The fibrotic area was stained blue in the images. Compared to the AMI group, there was more severe myocardial damage in the HL-AMI group (Figure 5).

Our data showed that, after AMI, the severity of myocardial injury might correlate with the level of blood lipids, hyperlipidemia may aggravate AMI-induced cardiac injury, and impede its repair.

### Hyperlipidemia attenuated the mobilization of EPCs stimulated by AMI

**Figure 6:**
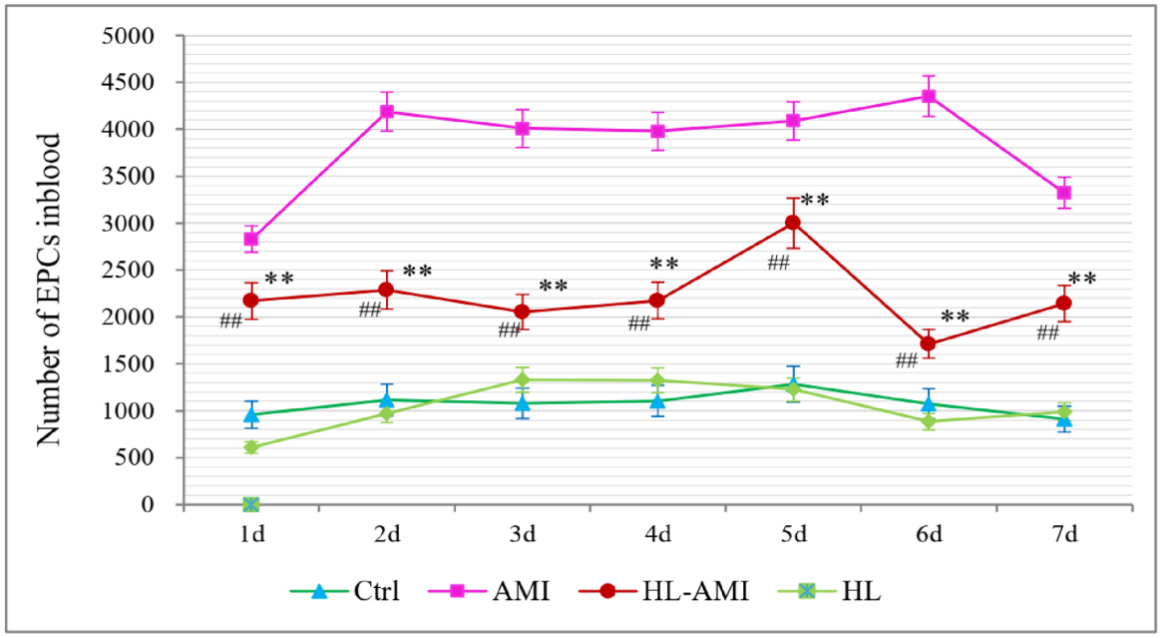
**Change in the number of CEPCs in each group rats during 7 days after AMI.**^ **^, *P* < 0.01, compared with the AMI group; ^##^, *P* < 0.01, compared with the HL group. Each data point is the average of the group of eight rats, and vertical lines indicate the SEM.

As shown in Figure 5, in the Ctrl and HL group, there is no significant change that could be observed in the count of CEPCs per mL during 7 days following AMI. The values were in the range 960–1330. In the AMI group, that count increased rapidly, reached its peak value (4353 ± 284/mL) on the 2nd day, and remained at this level to the 6th day, then decreased slowly. In the HL-AMI group, this number increased gradually, reached its maximum on the 5th day (3000 ± 374/mL), then began to decrease. However, the overall level and the maximum of CEPCs count were significantly lower than the AMI group (Figure 6, *P* < 0.05 or *P* < 0.01).

**Figure 7:**
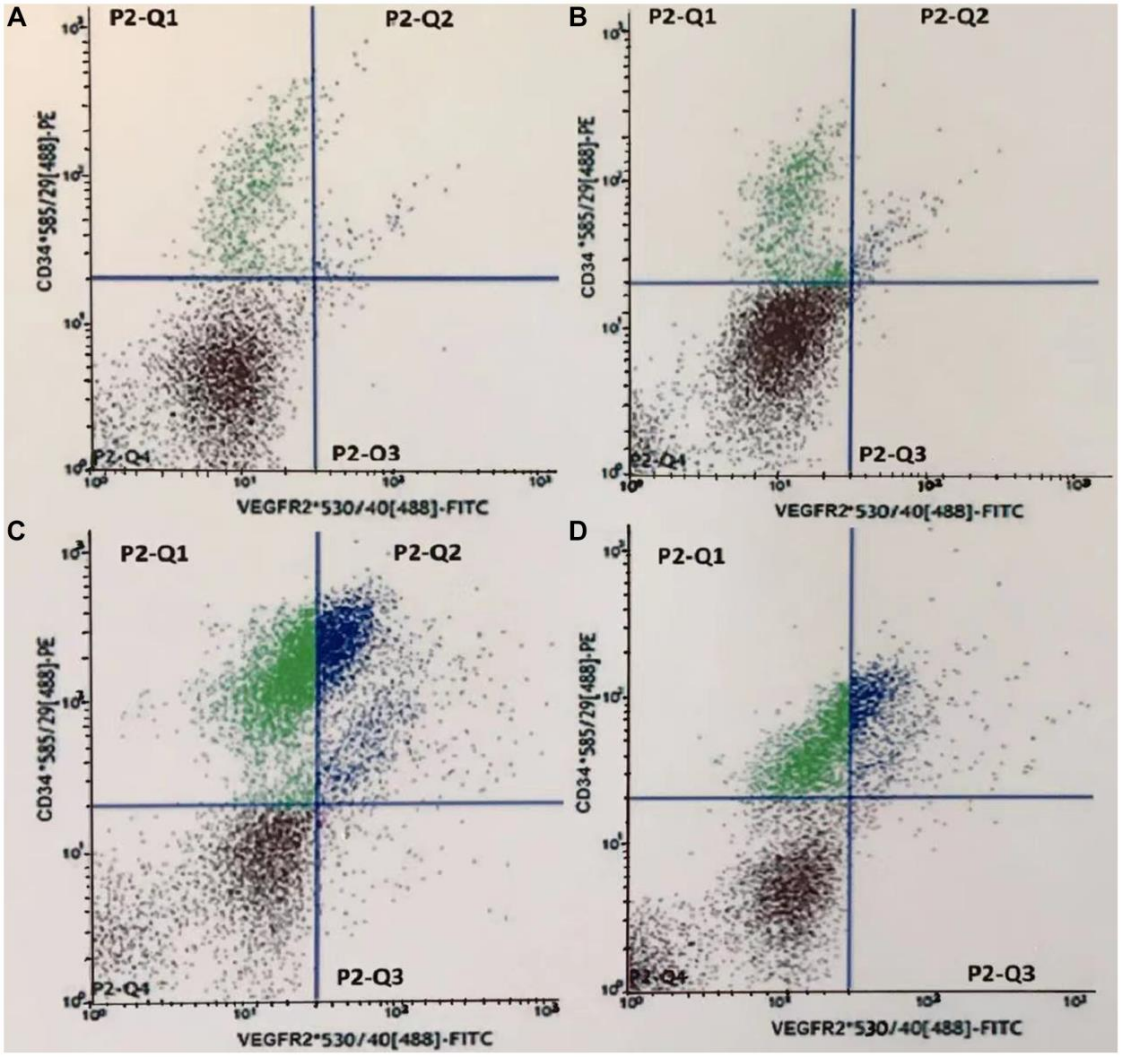
**Fluorescence intensity scatter diagrams of CEPCs in the 4th subgroup rats.** (**A**–**D**) represents the Ctrl, HL, AMI, and HL-AMI group, respectively. Cells in gate P2-Q2 (PE^+^/FITC^+^ or CD34^+^/VEGFR2^+^) are CEPCs.

The fluorescence intensity scatter diagrams of CEPCs in the 4th subgroup were shown in Figure 2. Dots in the P2-Q2 quadrant represent EPCs or CD34^+^/VEGFR2^+^ cells. This figure showed that, on the 4th day after AMI, the count of CEPCs in the HL-AMI group was significantly lower than the AMI group (Figure 7A–7D).

### Hyperlipidemia reduced the plasma levels of MMP-9, NO, eNOS, and VEGF in HL-AMI rats

**Figure 8:**
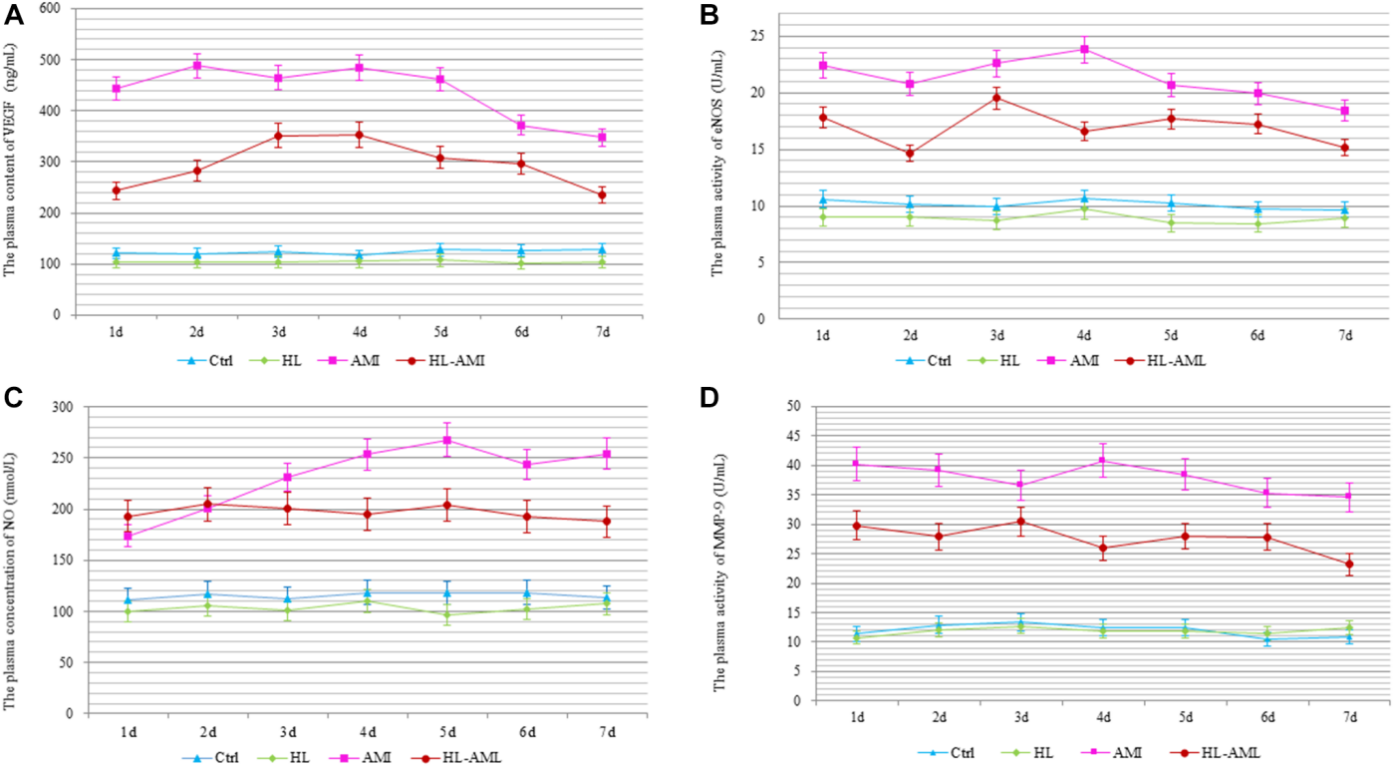
**Change in the plasma levels of MMP-9, NO, eNOS, and VEGF in each group rats during 7 days after AMI.** The Plasma content or activity of VEGF (**A**), eNOS (**B**), NO (**C**), and MMP-9 (**D**) in the Ctrl, HL, AMI, and HL-AMI group. ^*^, ^**^, *P* < 0.05 or *P* < 0.01, respectively, compared with the AMI group; ^#^, ^##^, *P* < 0.05 or *P* < 0.01, respectively, compared with the HL group. Each data point represents the average of eight rats in this group, and vertical lines indicate the SEM.

Within 7 days after the AMI operation, change in the plasma level of MMP-9, NO, eNOS and VEGF in four groups was shown in Figure 6. In the Ctrl and the HL groups, the plasma content and activity of these four cytokines fluctuated around a baseline level with no significant changes. Between the HL-AMI and AMI group, there was a similar variation trend that could be observed in this plasma content, which increased rapidly, reached the peak value, and then decreased gradually. However, compared with the AMI group, in the HL-AMI group, both the peak value and the whole level of above four cytokines plasma concentration all always remained at the lower level (Figure 8A–8D, *P* < 0.05 or *P* < 0.01).

### Hyperlipidemia down-regulated the expression of VEGF, eNOS and MMP-9 in myocardial tissue of the HL-AMI rats

**Figure 9:**
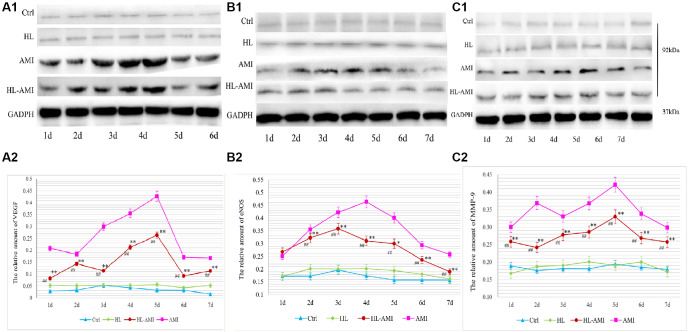
**VEGF, eNOS, and MMP-9 expression in the myocardium of each group rats during 7 days after AMI.** Western blot analysis of VEGF (**A1**), eNOS (**B1**), and MMP-9 (**C1**) protein expression in the myocardial tissue of the Ctrl, HL, AMI, and HL-AMI groups. GAPDH was used as the internal reference protein. (**A2**), (**B2**), and (**C2**) show the quantitative analysis of VEGF, eNOS, and MMP-9 respectively. ^*^, ^**^, *P* < 0.05 or *P* < 0.01, respectively, compared with the AMI group; ^#^, ^##^, *P* < 0.05 or *P* < 0.01, respectively, compared with the HL group. Each data point represents the average of the group of eight rats, and vertical lines indicate the SEM.

The VEGF, eNOS, and MMP-9 expression in the myocardial tissue within 7 days after the AMI operation was determined by western blotting (Figure 9).

In the Ctrl and the HL group rats, the expression of these proteins remained at a baseline level. Moreover, there were no significant differences between the two groups (Figure 9A1–9C2, *P* < 0.01).

In the AMI and HL-AMI groups, the change pattern in the expression of VEGF in myocardial tissue were similar. This expression increased significantly, reached its maximum on day 4, and then decreased quickly. However, the expression of this growth factor was lower in the HL-AMI group (Figure 9A1, 9A2, *P* < 0.01).

In the AMI group, the expression of eNOS was upregulated rapidly, reached its maximum on day 4, and then decreased gradually. In the HL-AMI group, that expression increased slowly, reached its maximum on day 2, and then began to decrease. However, the expression was lower (Figure 9B1, 9B2, *P* < 0.05 or *P* < 0.01).

In the AMI group, the changing trend in the expression of MMP-9 were more complicated. It was upregulated rapidly, reached its first peak value on day 2 and began to decrease, then reached another higher peak on day 5. In the HL-AMI group, the expression of this enzyme increased slowly and reached its maximum on day 5. The maximum and the overall expression remained significantly lower (Figure 9C1, 9C2, *P* < 0.05 or *P* < 0.01).

In summary, our results showed that, during 7 days after AMI, compared with the AMI rats, in the HL-AMI rats, the myocardial infarct size, the plasma activity of myocardial enzyme, and the degree of myocardial fibrosis all remained at the higher levels; meanwhile, the quantity of CEPCs, the expression and the plasma levels of VEGF, eNOS, NO, MMP-9 in myocardial tissue all decreased more significantly.

## DISCUSSION

For various CVDs, hyperlipidemia is not only one of the main risk factors, but also related to the worse prognosis. It is reported that hyperlipidemia may lead to CVDs through attenuating the adhesion, migration, and tube formation of EPCs, and accelerated its senescence [11, 12]. In this study, we first found that, after the same AMI modeling operation, compared with the normal rats, in the hyperlipidemia rats, the quantity of CEPCs increased more slowly, the peak value came later, the overall level and the maximum count of this cells all remained at the significantly lower level. Our results directly demonstrated that hyperlipidemia significantly reduced the mobilization of BM EPCs induced by AMI.

Our data also showed that, compared with the normal rats, in the hyperlipidemia rats, the MI size, the plasma activity of myocardial enzyme, and the myocardial fibrosis all remained at the higher level after AMI. This result indicated that hyperlipidemia significantly hindered the repair of cardiac injury induced by AMI. In view of the important role of EPCs in cardiac damage repair following AMI, the down-regulation of EPCs mobilization might be partly responsible for the worse myocardial injury repair in the hyperlipidemia animals.

After AMI, ischemic myocardial tissue releases a number of substances, including MMP-9, NO, eNOS, and VEGF, which play crucial roles in mobilizing BM EPCs [8, 10]. MMP-9 transforms the stem cell factor receptor (Kit) from the membrane-bound state into the soluble state, which converts BM EPCs from the quiescent state to the proliferative state, and allows them to migrate to peripheral blood [16–18]. MMP-9 is activated by VEGF via the VEGF/eNOS/NO/MMP-9 signal pathway [8, 19–21]. Banerjee S reported that, in familial hyperlipidemia acute coronary syndrome patients that had been treated with intensive lipid-lowering therapy for 90 days, accompanied by the significant decline in their plasma lipids, there was a robust mobilization of EPCs colony-forming unit from baseline [22]. Lin LY found that, in patients with hypercholesterolemia and type 2 diabetes, after were treated by lipid-lowering drug, the counts of CEPCs, the expression and phosphorylation of eNOS in these cells were all increased, moreover, the plasma level of VEGF in these patients was also up-regulated [23]. Their results indirectly indicated that hyperlipidemia might impair the mobilization of EPCs, and might downregulate the expression of VEGF and eNOS. Our results directly attested that hyperlipidemia significantly inhibited the expression and the secretion of the MMP-9, NO, eNOS, and VEGF in myocardial tissue, which may account for that hyperlipidemia prevents EPCs mobilization stimulated by AMI.

In previous studies, we characterized the kinetics pattern of CEPCs count, the daily changing mode on the plasma level of VEGF etc., in normal animals within 7 days following AMI [8]. These changing curves in hyperlipidemia animal were further graphed in the present study. In our study, rats in each treatment group were further subdivided into 7 subgroups by the date following modeling. At serial time points after AMI, the serial subgroups of rats were phlebotomized from their celiac artery in turn, they suffered no hemorrhagic injury before sacrifice except the coronary artery ligation itself. Our ingenious method could maximally exclude other interferences to provide an objective reflection for the mobilization of BM EPCs stimulated by AMI.

Our results provide new insights into the relationship among the hyperlipidemia, the mobilization of EPCs, and the repair of heart damage induced by AMI; this relationship may partly account for that hyperlipidemia patients have worse prognosis after AMI. In our following studies, the molecular mechanism underlying the potential interaction of hyperlipidemia and EPCs during AMI.
